# Evolution of the Electronic Structure of Hybrid Organic–Inorganic Halide Perovskite Single Crystals upon Alkali Metal Doping

**DOI:** 10.1021/acs.jpclett.6c01932

**Published:** 2026-07-29

**Authors:** Jinpeng Yang, Tingyu Jiao, Wei Li, Bo Xu, Mats Fahlman, Satoshi Kera, Xianjie Liu

**Affiliations:** † College of Physical Science and Technology, 38043 Yangzhou University, Jiangsu 225009, China; ‡ National Key Laboratory for Metrology and Calibration Techniques, 38041 China Institute of Atomic Energy, Beijing 102413, China; § School of Science, 56651 China Pharmaceutical University, Nanjing, 211198, China; ∥ Laboratory for Organic Electronics, Department of Science and Technology, 4566 Linköping University, Norrköping SE-60174, Sweden; ⊥ 88301 Institute for Molecular Science, Myodaiji, Okazaki 444-8585, Japan

## Abstract

Alkali-metal incorporation is widely employed to improve the performance and stability of organic halide perovskites, yet the underlying doping mechanisms and diffusion pathways remain poorly understood. Here, we use ultraviolet photoemission spectroscopy to probe the evolution of the electronic structure of CH_3_NH_3_PbX_3_ (X = I, Br, and Cl) single crystals upon potassium and cesium deposition. We observe two distinct regimes governed by halide chemistry: facile bulk diffusion in CH_3_NH_3_PbI_3_ and CH_3_NH_3_PbBr_3_, in contrast to surface-confined accumulation in CH_3_NH_3_PbCl_3_, arising from kinetically hindered alkali migration in the chloride perovskite. Temperature-dependent measurements of the doped crystals further detect opposite valence band shifts for CH_3_NH_3_PbBr_3_ relative to CH_3_NH_3_PbI_3_ and CH_3_NH_3_PbCl_3_, highlighting the role of surface termination and charge redistribution. Notably, alkali metal deposition induces substantial energy level shifts while leaving band dispersions largely unchanged, indicating a predominantly interfacial doping mechanism. These results establish a direct link among halide composition, diffusion behavior, and electronic structure evolution, providing microscopic insight into alkali-metal interactions in perovskites.

## Graphical abstract

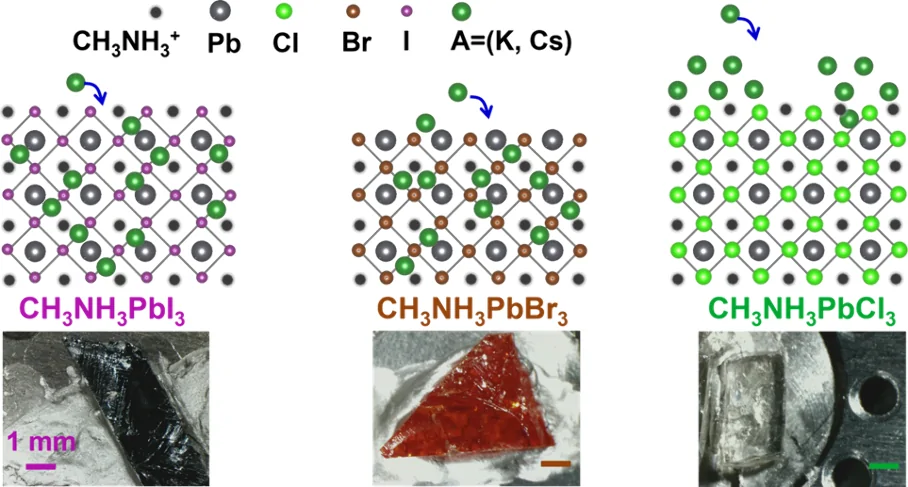

Hybrid organic–inorganic perovskite solar cells (PSCs) with a single junction have already shown better device performance with power conversion efficiencies reaching 27% due to their remarkable optoelectronic properties, including a high light absorption coefficient, high charge carrier mobility/lifetime, long diffusion length, low exciton binding energy, and low-cost film processing.^1–5^ The superior features of such materials make PSCs very promising for the future cutting-edge photovoltaic techniques particularly using related tandem structures with large-scale commercialization. However, there is still an urgent need to enhance the lifetime and stability of PSCs, which commonly require, e.g., additional surface modification and different film growth methods.^6–9^ On the other hand, alkali metals have been shown to play a crucial role in improving the crystal structures as well as stabilize the intrinsic properties of hybrid perovskites.^10–13^ In addition, they can act as n-type dopants, enabling interfacial energy-level tuning and more efficient charge-carrier transport in practical devices. More generally, alkali metals can compensate halide-vacancy-related defects by immobilizing excess halides through formation of benign alkali-halide species at grain boundaries and interfaces, thereby improving PSCs stability.^14–16^ However, the underlying mechanism of diffusion processes and the efficacy of alkali metal doping are still only partially understood.

Perovskite single crystals offer key advantages compared to polycrystalline thin films, such as reduced grain boundaries that can effectively suppress ion migration and material degradation, as well as a low density of gap states,^17,18^ making them ideal platforms to probe their intrinsic electronic properties.^19–21^ In particular, their effectively infinite grain size enables quantitative studies of electronic structure evolution during alkali-metal deposition and diffusion from the top perovskite surfaces, which are often hindered in conventional polycrystalline films due to grain-boundary-limited doped penetration.^22^ Here, we investigate the doping properties using cleaved single crystal surfaces to minimize the influence of grain boundaries and to effectively eliminate potential disruptions caused by structural defects inherent to perovskites.

In this study, we conducted a comprehensive investigation of the electronic structure evolution in hybrid organic–inorganic halide perovskite single crystals CH_3_NH_3_PbX_3_ (X = I, Br, Cl) upon doping with alkali metals (K and Cs) by combining interfacial techniques, including ultraviolet photoemission spectroscopy (UPS), angle-resolved photoemission spectroscopy (ARPES), and atomic force microscopy (AFM). We observe pronounced differences in alkali-metal deposition-induced diffusion, characterized by atomic penetration from the surface into the bulk. Both K and Cs readily diffuse in iodide- and bromide-based perovskites, while their diffusion is significantly hindered in the presence of chloride ions. During continuous deposition, alkali metals accumulated at the top surface of CH_3_NH_3_PbCl_3_, eventually forming metallic layers or clusters, as evidenced by the emergence of a step-like Fermi edge in UPS. Furthermore, temperature-dependent measurements on both alkali-metal-doped and pristine perovskite single crystals demonstrate different shifts in the valence band maxima and work function shifts. These results suggest different surface terminations: CH_3_NH_3_PbI_3_ and CH_3_NH_3_PbCl_3_ are likely terminated by a CH_3_NH_3_^+^-X plane, whereas CH_3_NH_3_PbBr_3_ is Pb-Br terminated.

Figure 1 presents the evolution of UPS spectra for three different perovskite single crystals as a function of the evaporated K (Cs) thickness, highlighting the effects on both the secondary electron cutoff (SECO) and valence band regions. These spectra correspond to CH_3_NH_3_PbI_3_ (a and d), CH_3_NH_3_PbBr_3_ (b, e), and CH_3_NH_3_PbCl_3_ (c, f) with K and Cs doping, respectively. The UPS valence band feature of pristine perovskite single crystals (0 Å) is consistent with previous reports.^23–26^ The extracted ionization energies (IEs), obtained from the sum of the work function (WF) and valence band maximum (VBM) for six different crystal samples, are 5.58 and 5.80 eV for CH_3_NH_3_PbI_3_, 6.16 and 6.10 eV for CH_3_NH_3_PbBr_3_, and 7.17 and 7.15 eV for CH_3_NH_3_PbCl_3_. Notably, CH_3_NH_3_PbI_3_ exhibits a relatively larger energy variation (∼0.2 eV) compared to the smaller differences (<0.1 eV) observed in CH_3_NH_3_PbBr_3_ and CH_3_NH_3_PbCl_3_ crystals, likely reflecting the variations in surface quality arising from crystal growth and sample preparation, such as a knife-cutting process. No film charging is detected at room temperature, as evidenced by the sharp SECO features in Figure 1 and the well-defined band dispersions presented below. Upon K atoms deposited onto CH_3_NH_3_PbI_3_ (Figure 1(a)) and CH_3_NH_3_PbBr_3_ (Figure 1(b)) crystal surfaces, both the SECO and VBMs progressively shift toward higher binding energies with increasing K content. Concurrently, new spectral features gradually emerge in the valence band region, as highlighted by the down arrow in Figure 1(a). Initially, the broader features from pristine CH_3_NH_3_PbI_3_ become gradually sharper upon K deposition. Two peaks are observed at the binding energies of 3.93 and 5.28 eV. Moreover, no additional peak or tail states above the VBM edge in either CH_3_NH_3_PbI_3_ or CH_3_NH_3_PbBr_3_ are detectable (see insets of Figure 1(a,b) and Figure S1 in Supporting Information), indicating the absence of gap states upon K deposition. It has been observed that K doping usually introduces significant spectral broadening because the existing structural defects and grain boundaries in thin films can hinder uniform dopant diffusion.^27–30^ However, in our perovskite single crystals, the absence of these defects enables more uniform alkali metal atom distribution to suppress the spectral broadening. Due to the negligible VBMs shifts, the well-defined spectral features from K doping, the relatively sharper SECO feature (particularly in Figure 1(a,b)), and the clearly observed band dispersion (see following discussion), we can propose that K deposition does not significantly alter the surface morphology of the perovskite single crystals. Moreover, no K atom aggregation is observed at the surface. Instead, K atoms are likely to somehow diffuse from the surface into the bulk, such behavior is consistent with theoretical predictions and experimental observations.^14,27,31^ In contrast, K-deposited CH_3_NH_3_PbCl_3_ crystal exhibits a different behavior, as shown in Figure 1(c). Unlike the pronounced and gradual shift of both VBM and SECO toward higher binding energies observed in Figure 1(a,b), only a minor energy shift (∼0.15 eV) is detected in Figure 1(c). Furthermore, a step-like feature reaching the baseline at the Fermi level (binding energy is 0 eV) is observed, as shown in the inset of Figure 1(c), and more detail presented in Figure S1c labeled with a red arrow. This indicates the formation of metallic clusters upon K deposition, accompanied by the emergence of additional gap states. This observation suggests that K atoms remain confined to the top surface of CH_3_NH_3_PbCl_3_ crystals without penetrating into the bulk. One plausible explanation could be the formation of an interfacial KCl layer at the top surface,^32^ where such a thin layer likely acts as a barrier to hinder further diffusion of K atoms into CH_3_NH_3_PbCl_3_.

**Figure 1:**
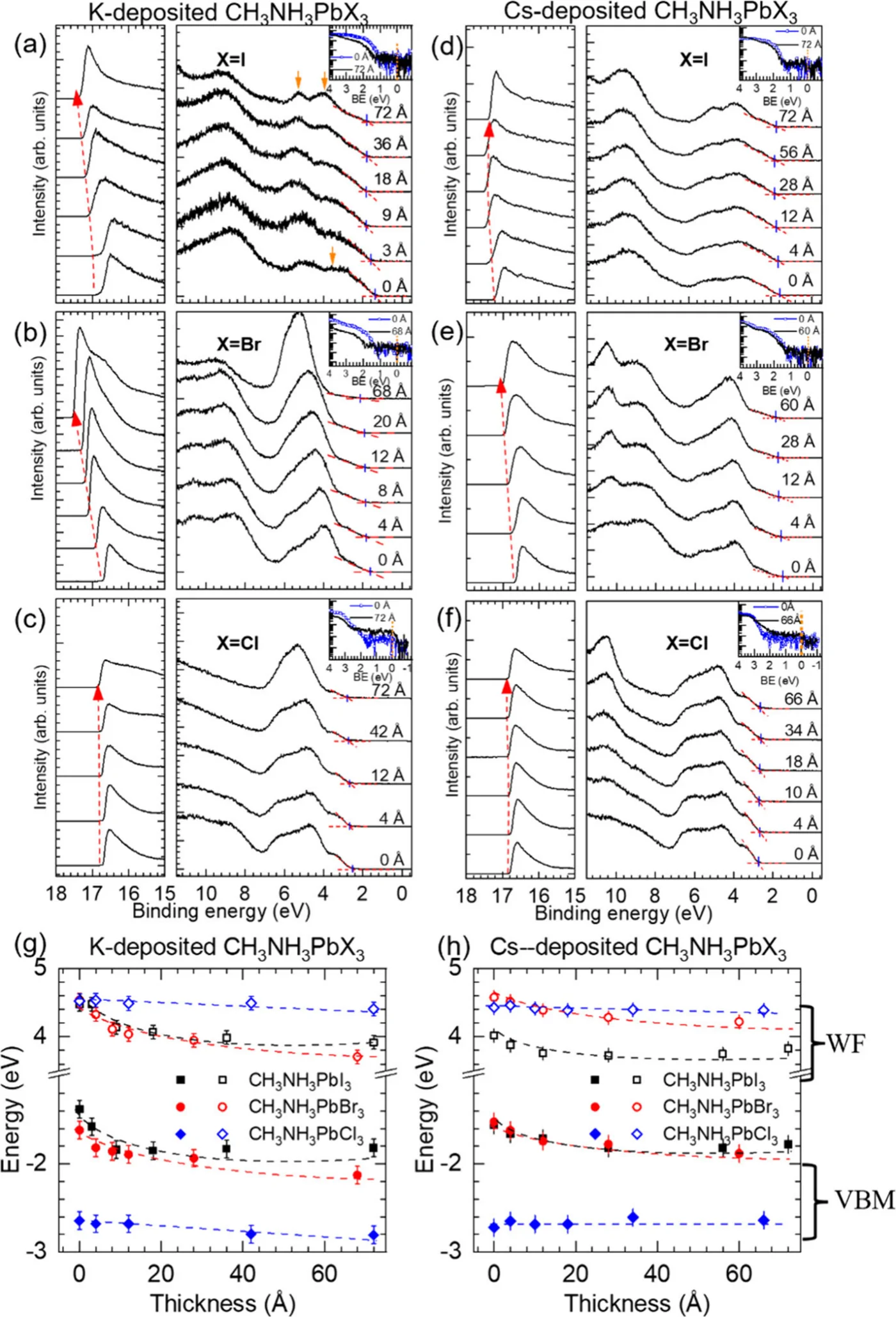
Evolution of UPS spectra of K- (a–c) and Cs- (d–f) deposited CH_3_NH_3_PbX_3_ single crystal surfaces as a function of incremental deposition thickness with X = I, Br, and Cl, where 0 Å indicates UPS spectra of the pristine crystal; the amplified spectrum close to the Fermi level (vertical dashed line) is shown in each inset. The red dashed arrows are guidelines of the change of SECO upon doping. Panels (g) and (h) summarize the energy shifts of WF (open symbols) and VBM for K- and Cs- deposited perovskite single crystals upon deposited K and Cs thicknesses.

When Cs is deposited on CH_3_NH_3_PbI_3_ and CH_3_NH_3_PbBr_3_ crystal surfaces (Figure 1(d) and (e)), both the VBM and SECO shift toward higher binding energies, similar to the behavior observed for K deposition. For example, two peaks appear at binding energies of 3.77 and 4.82 eV in Cs-deposited CH_3_NH_3_PbI_3_ (Figure 1(d)), while an additional feature at 10.48 eV emerges in Cs-deposited CH_3_NH_3_PbBr_3_ (Figure 1(e)), which can be attributed to the density of states derived from Cs 5p orbitals.^24,33^ These features indicate successful Cs doping. Meanwhile, no additional peak or tail states above the VBM are observed, as shown in the insets of Figure 1(d) and (e), which confirms that Cs atoms can diffuse from the surface into the bulk of these crystals. In contrast, Cs deposition on CH_3_NH_3_PbCl_3_ displays no noticeable shifts in either the VBMs or SECO, as shown in Figure 1(f). Increasing Cs coverage gives rise to a pronounced step-like Fermi edge (the inset of Figure 1(f)), suggesting that Cs atoms do not penetrate into the bulk of CH_3_NH_3_PbCl_3_. Instead, they remain localized at the surface and gradually form metallic clusters, reflecting the behavior previously observed for K-deposited CH_3_NH_3_PbCl_3_.

The extracted energy shifts in WFs and VBMs induced by alkali metals doping for all three perovskite single crystals are summarized in Figure 1(g) and (h), respectively, presenting two different trends at perovskite interfaces. For K-deposited CH_3_NH_3_PbI_3_ and CH_3_NH_3_PbBr_3_, the spectral features exhibit a gradual downshift of ∼0.5 eV with increasing K thickness. In contrast, K-deposited CH_3_NH_3_PbCl_3_ shows only a small linear shift (∼0.15 eV), as shown in Figure 1(g). These results suggest that K atoms effectively dope and diffuse into the bulk of CH_3_NH_3_PbI_3_ and CH_3_NH_3_PbBr_3_, whereas in CH_3_NH_3_PbCl_3_ they may remain confined to the top surface without bulk penetration. A similar trend is also observed for Cs-deposited perovskite single crystals; the spectral features of doped CH_3_NH_3_PbI_3_ and CH_3_NH_3_PbBr_3_ display a gradual downward shift with increasing Cs thickness, whereas deposited CH_3_NH_3_PbCl_3_ exhibits negligible change, as illustrated in Figure 1(h). Despite their different ionic sizes, both K and Cs remain strongly surface-enriched in CH_3_NH_3_PbCl_3_. The observed K (Cs) on CH_3_NH_3_PbCl_3_ is consistent with a proposed model of the formation of an interfacial KCl (CsCl) layer, together with the stronger Pb–Cl bonding and higher migration barriers, which could collectively suppress alkali-ion migration, confining the dopants mainly to the surface/interface region. Further complementary structural or depth-profiling studies will be helpful to verify the model.

Next, we examine temperature effects on doped CH_3_NH_3_PbX_3_ crystal surfaces to reveal intrinsic doping-induced electronic changes from thermal effects. Figure 2 illustrates the UPS evolution for K- and Cs-doped perovskite single crystals upon cooling from 300 to 100 K. The initial UPS spectra of K, Cs-deposited samples at 300 K correspond to the highest deposition levels in Figure 1. Upon decreasing temperature below 250 K, the VBMs and SECO slowly shift toward the higher binding energies in K-deposited CH_3_NH_3_PbI_3_ crystals, as shown in Figure 2(a). Moreover, the spectral features become significantly clearer at lower temperatures, with enhanced peak definition compared to 300 K, as indicated by the pink down arrows. A similar upshift of both the VBM and SECO is also observed in K-deposited CH_3_NH_3_PbCl_3_, as shown in Figure 2(c). The broadening of the SECO in CH_3_NH_3_PbCl_3_ crystals at lower temperatures indicates a slight charging effect, which can be attributed to (i) the formation of discontinuous K clusters rather than a uniform film, and (ii) the relatively large thickness of the CH_3_NH_3_PbCl_3_ crystals combined with reduced conductivity at low temperatures.^34^ In contrast, a different behavior is observed in K-doped CH_3_NH_3_PbBr_3_ crystals. Upon cooling to 100 K, both the VBM and SECO shift toward lower binding energies, accompanied by better resolved spectral features, as shown in Figure 2(b). For Cs-deposited CH_3_NH_3_PbX_3_ single crystals, similar temperature-induced band structure evolutions are observed, as shown in Figure 2(d)–(f). In Cs-deposited CH_3_NH_3_PbI_3_ crystal, both the VBM and SECO upshift about 0.1 eV upon cooling. A comparable upshift is also evident in Cs-doped CH_3_NH_3_PbCl_3_ crystals upon temperature, as shown in Figure 2(f). Conversely, the Cs-deposited CH_3_NH_3_PbBr_3_ crystal exhibits a gradually rigid shift of the entire spectrum toward the lower binding energies during reducing temperatures. Meanwhile, clearer band structures with better resolved peaks, marked by the pink down arrows, can be observed in Figure 2(e). It is worth noting that similar temperature-dependent shifts of the VBM and SECO are also observed in pristine (undoped) CH_3_NH_3_PbX_3_ perovskite single crystals (Figure S2). In pristine CH_3_NH_3_PbI_3_ and CH_3_NH_3_PbCl_3_, both UPS spectra exhibit an upward shift with decreasing temperature, whereas a downward shift is observed in CH_3_NH_3_PbBr_3_. Furthermore, repeated UPS measurements after returning the samples to near room temperature reveal no significant spectral changes (Figure S3), confirming the reversibility of these temperature-induced effects.

**Figure 2:**
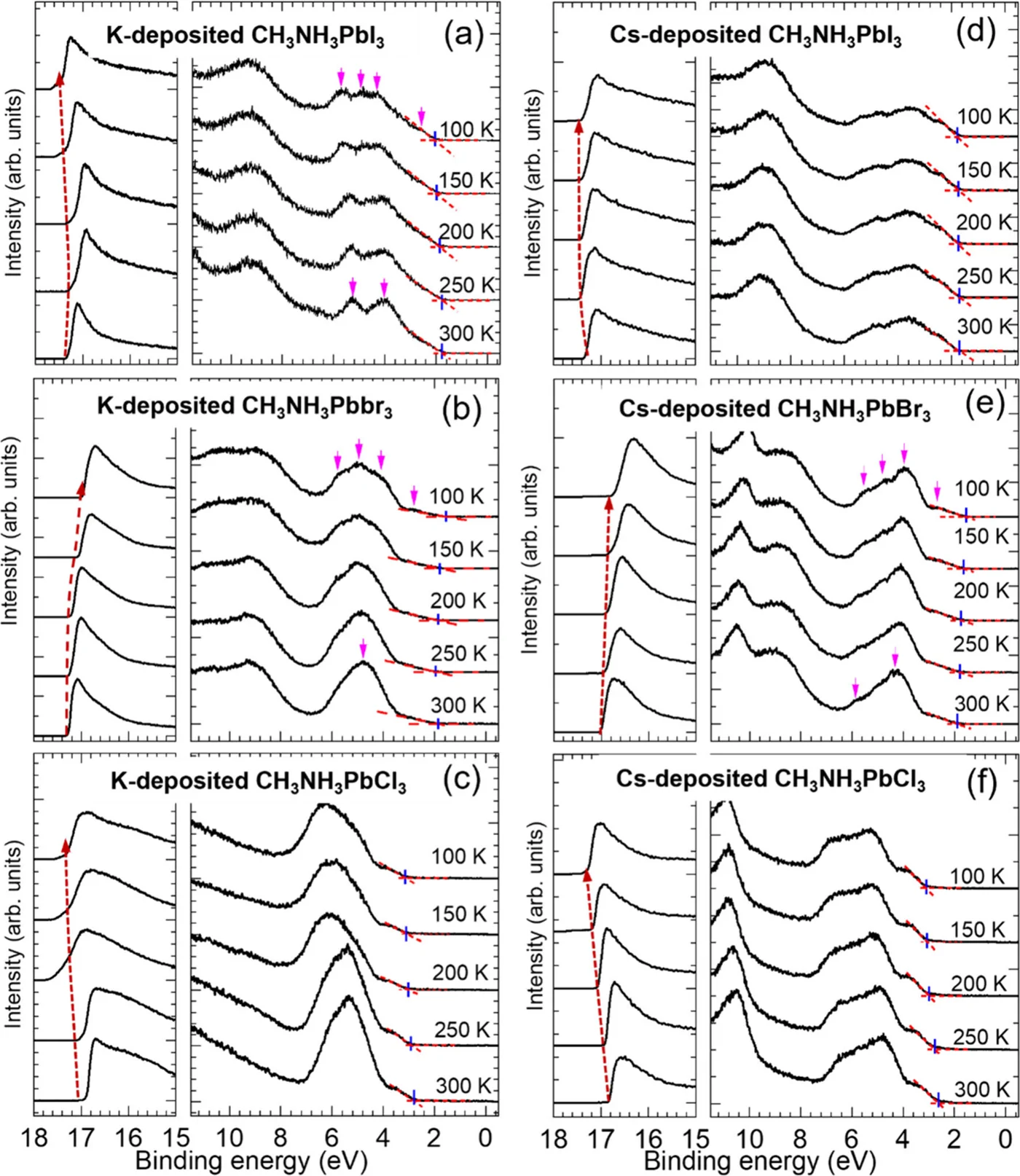
Temperature-dependent UPS spectra of (a–c) K- and (d–f) Cs- deposited CH_3_NH_3_PbX_3_ single crystals with X = I, Br, and Cl from top to bottom, respectively. The starting spectra at 300 K are with the highest K (Cs)- deposition thickness from Figure 1, followed by cooling to 100 K, as labeled in each spectrum. The VBM position is indicated with the vertical blue bar. The pink down arrows in (a), (b), and (e) indicate the clearly resolved spectral features. The red dashed arrows are guidelines for the change of SECO upon temperature.

Figure 3 summarizes the energy shifts in WF and VBM for both alkali metal-surface-doped and pristine perovskite single crystals as a function of temperature. The energy values for the doped samples are directly extracted from the UPS data in Figure 2. The temperature-induced changes in the VBMs and WFs follow two clear trends: (i) Both the VBMs and WFs exhibit an approximately linear dependence on temperature across all samples. (ii) In both doped and pristine CH_3_NH_3_PbI_3_ and CH_3_NH_3_PbCl_3_ crystals, the WF and VBM decrease linearly as the temperature decreases. However, CH_3_NH_3_PbBr_3_ crystals exhibit the opposite behavior, with WFs and VBM increasing linearly as the temperature is reduced. Importantly, this opposite energy shift cannot be attributed to differences in alkali-metal deposition behavior (i.e., bulk diffusion versus surface accumulation) since the characteristic band structure evolution induced by alkali-metal diffusion has been verified in both CH_3_NH_3_PbI_3_ and CH_3_NH_3_PbBr_3_ (see Figure 1). We attribute these differences to opposite surface dipoles arising from different surface terminations, based on the following considerations: (i) Opposing dipoles typically result in contrasting valence band shifts in temperature-dependent experiments—for example, reversed surface dipoles in pentacene and perfluoropentacene lead to opposite band shifts upon cooling.^35^ (ii) In hybrid perovskites, Pb-X terminations induce a downward surface dipole, whereas CH_3_NH_3_-X terminations generate an upward dipole.^36^ (iii) Alkali metal deposition has a limited influence on the surface dipole, as deposited atoms predominantly diffuse into the bulk. Moreover, MAES (meta-atom electron spectroscopy) results for CH_3_NH_3_PbI_3_ and CH_3_NH_3_PbBr_3_ crystals further support different surface terminations (Figure S4).^37^

**Figure 3:**
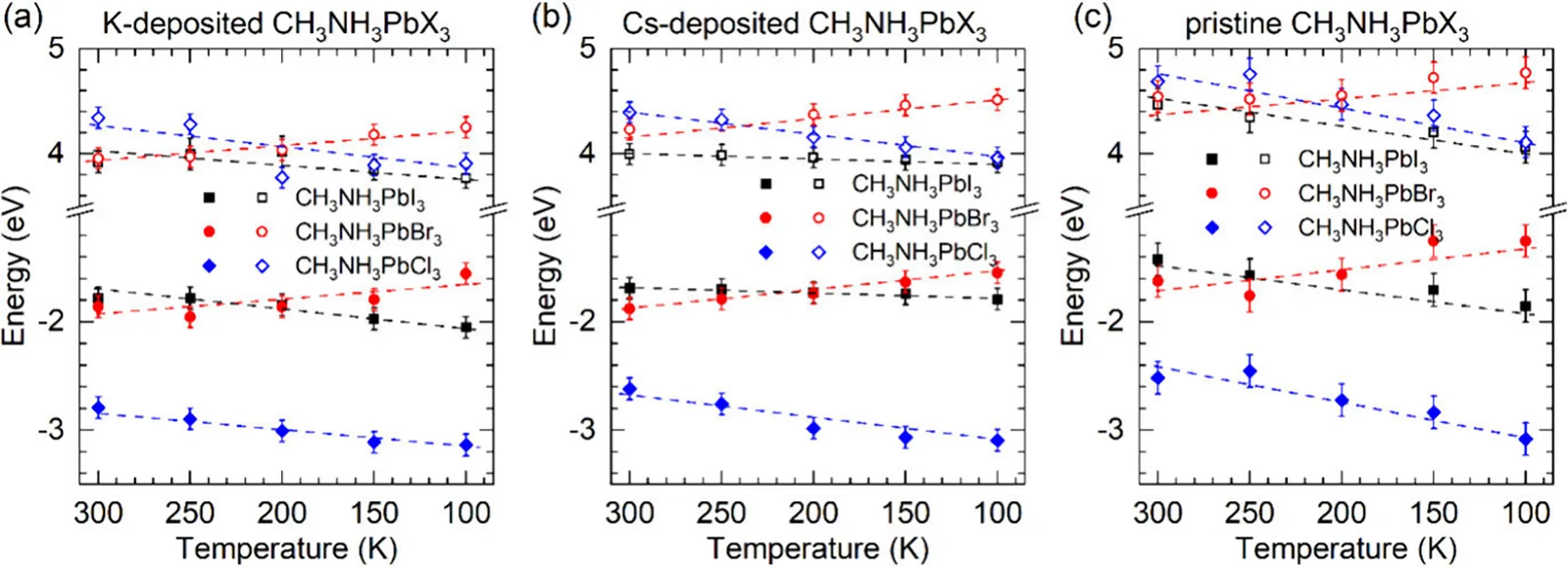
Energy shifts of the WFs (open symbols) and VBM (filled symbols) of (a) K-deposited, (b) Cs- deposited, and (c) pristine perovskite single crystals CH_3_NH_3_PbX_3_ (X = I, Br and Cl) as a function of the measured temperatures.

To further investigate the influence of alkali metal deposition on the surface geometries of perovskite single crystals, we performed ARPES measurements along the cubic Γ^C^M^C^ (ΓM) direction for both CH_3_NH_3_PbI_3_ and CH_3_NH_3_PbBr_3_ as 21.22 eV photon energy (He I_α_ light) can only access the ΓM direction in CH_3_NH_3_PbX_3_.^21,38^ Figure 4 presents the ARPES results for pristine and K-doped crystals at different temperatures. Detailed band dispersion measurements, highlighting the underlying crystal periodicities, are provided in Figure S5. In contrast, no clear band dispersion can be observed even in pristine CH_3_NH_3_PbCl_3_ crystals, as shown in Figure S6, most likely arising from its unique surface morphology, which exhibit significantly smaller flat terraces with highly dense step bunching than other two perovskite single crystals, as revealed by AFM (Figure S7). Here we primarily focus on ARPES results obtained upon the K deposition case, as Cs-doped CH_3_NH_3_PbI_3_ and CH_3_NH_3_PbBr_3_ show similar spectral behavior (as shown in Figure S8). ARPES data of pristine CH_3_NH_3_PbI_3_ show a clear band dispersion of the top valence band (VB) along the ΓM direction at 300 K in Figure 4(a). Near the Γ point, the top VB appears as a flat band, while a significant dispersion is observed at the M point (horizontal red dashed line), with a dispersion periodicity of 2 × 0.70 Å^–1^ and a total energy shift of approximately 0.7 eV. These observations are consistent with reported ARPES results^39^ which indicate that the CH_3_NH_3_PbI_3_ surface adopts a cubic-like phase at 300 K.^34,40^ For 72 Å K-deposited CH_3_NH_3_PbI_3_ crystal, the ARPES results reveal that the band dispersion along the ΓM direction remains clearly preserved (middle panel of Figure 4(a)), indicating that K diffusion into the bulk does not disrupt the surface crystalline structure. However, a clear shift of ∼0.4 eV at M point away from the Fermi edge (red dashed line) is observed. Furthermore, no evidence of phase transition is detected in the surface region of the K-deposited CH_3_NH_3_PbI_3_ crystal during low-temperature measurements. The right panel of Figure 4(a) shows the ARPES data at 100 K for the 72 Å K-deposited CH_3_NH_3_PbI_3_ crystal. The band dispersion along the ΓM direction remains nearly unchanged compared to that at 300 K, with the exception of a rigid shift of the entire band structure toward higher binding energy.

**Figure 4:**
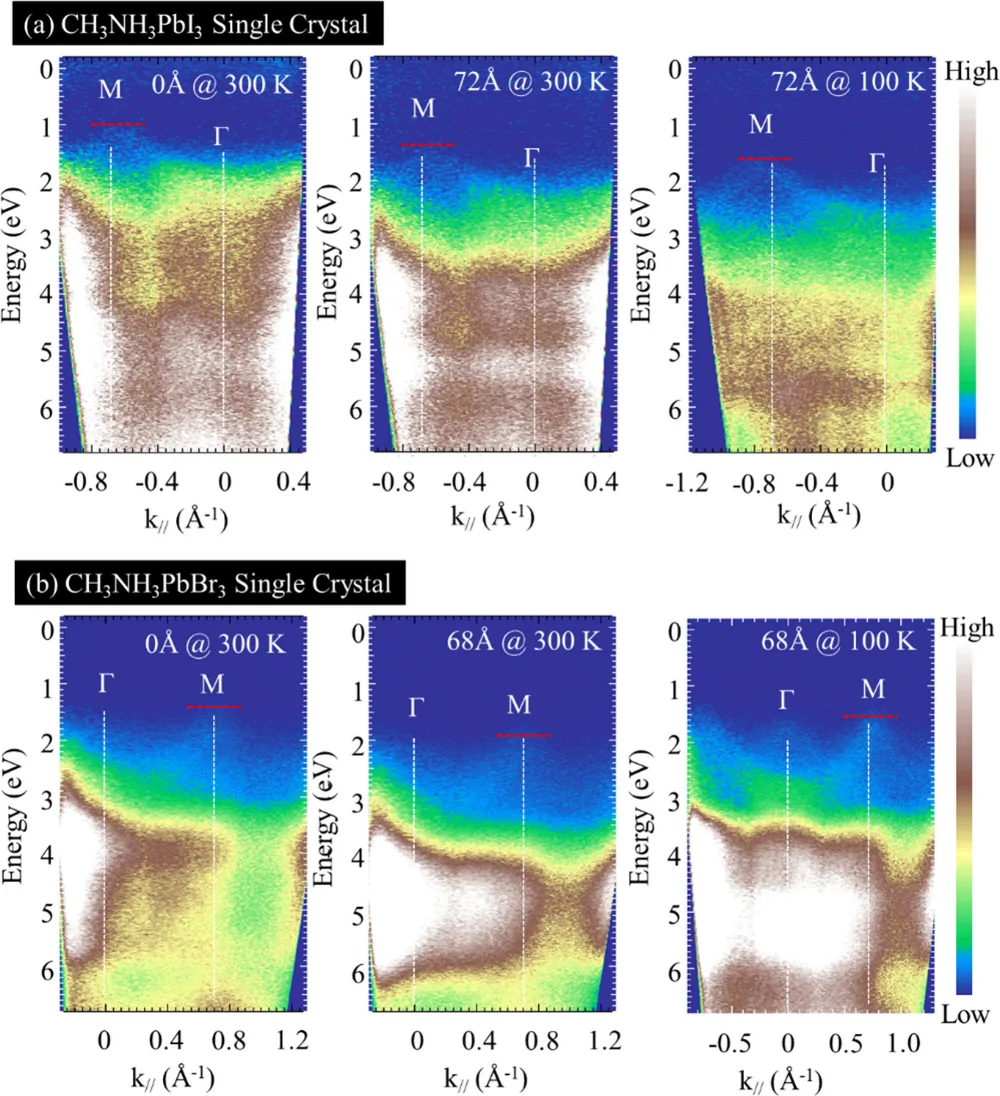
Band structure of the perovskite single crystals with and without doping under different temperatures. He I_α_-ARPES spectra of (a) CH_3_NH_3_PbI_3_ and (b) CH_3_NH_3_PbBr_3_ single crystal along a cubic ΓM direction. The left panel represents the pristine perovskite single crystal spectrum at 300 K; the middle panel shows the influence of electronic structure on the highest K doping at 300 K; the right panel depicts the spectrum of surface-doped crystals measured at 100 K.

Figure 4(b) shows the band dispersions of CH_3_NH_3_PbBr_3_ single crystals upon K deposition at different temperatures, where the ARPES spectra reveal significant changes in the top VB dispersions compared to CH_3_NH_3_PbI_3_. For pristine CH_3_NH_3_PbBr_3_ measured at 300 K (left panel of Figure 4(b)), a clear band dispersion of the top VB can be observed with a maximum at the M point (∼0.75 Å^–1^).^37^ Upon 68 Å K deposition, CH_3_NH_3_PbBr_3_, the band dispersion along the ΓM direction remains largely unchanged (middle panel of Figure 4(b)), except for a gradual shift of the entire band structure toward higher binding energy. However, an additional top VB appears at the Γ point upon K deposition, becoming more pronounced and clearly resolved at 100 K (right panel of Figure 4(b)). This results in a reduction of the momentum-space periodicity from ∼2 × 0.75 to ∼2 × 0.38 Å^–1^. The change indicates the occurrence of a phase transition, not only in the pristine perovskite crystal surface,^37^ but also in the K- deposited surface. Furthermore, an opposite shift in the band structure is clearly observed, with the VB moving toward lower binding energy (red dashed lines), in clear contrast to Figure 4(a). These observed differences between CH_3_NH_3_PbI_3_ and CH_3_NH_3_PbBr_3_ crystals indicate the presence of distinct surface structures, particularly at the topmost layer. Here, our observations can also be explained by the mechanism proposed in Figure 3, where the opposite VB shifts are attributed to differences in surface dipoles arising from distinct geometries and chemical elemental compositions and their different responses to low temperature due to charge redistribution. Specifically, the CH_3_NH_3_PbI_3_ topmost surface is likely terminated by a CH_3_NH_3_^+^-I plane, while CH_3_NH_3_PbBr_3_ is more likely terminated by a Pb-Br plane.^36,37,41^

The energy level diagrams of all investigated systems, before and after alkali metal deposition, are summarized schematically in Figure 5. Note that the conduction band minimum (CBM) (also electron affinity) cannot be directly accessed using UPS; therefore, published values from thin films are adopted: 1.7 eV for CH_3_NH_3_PbI_3_, 2.32 eV for CH_3_NH_3_PbBr_3_, and 3.09 eV for CH_3_NH_3_PbCl_3_, respectively.^23^ Figure 5(a) illustrates the diffusion processes and corresponding topmost surface structures for alkali metal deposition in the three perovskite single crystals. CH_3_NH_3_PbI_3_ and CH_3_NH_3_PbCl_3_ are terminated by CH_3_NH_3_^+^-X (X = I, Cl) planes, while CH_3_NH_3_PbBr_3_ is terminated by a Pb-Br plane. Upon alkali-metal deposition, K and Cs atoms can easily diffuse into the bulk of CH_3_NH_3_PbI_3_ and CH_3_NH_3_PbBr_3_ crystals. In contrast, in CH_3_NH_3_PbCl_3_, the alkali metals preferentially accumulate at the top surface, forming clusters. Figure 5(b) summarizes the evolution of the band structure upon alkali-metal deposition. In CH_3_NH_3_PbI_3_ and CH_3_NH_3_PbBr_3_, pronounced surface band bending develops with increasing alkali-metal deposition. In contrast, in CH_3_NH_3_PbCl_3_, surface accumulation of alkali metals causes only a slightly linear downward shift in the band structure due to the absence of effective doping.

**Figure 5:**
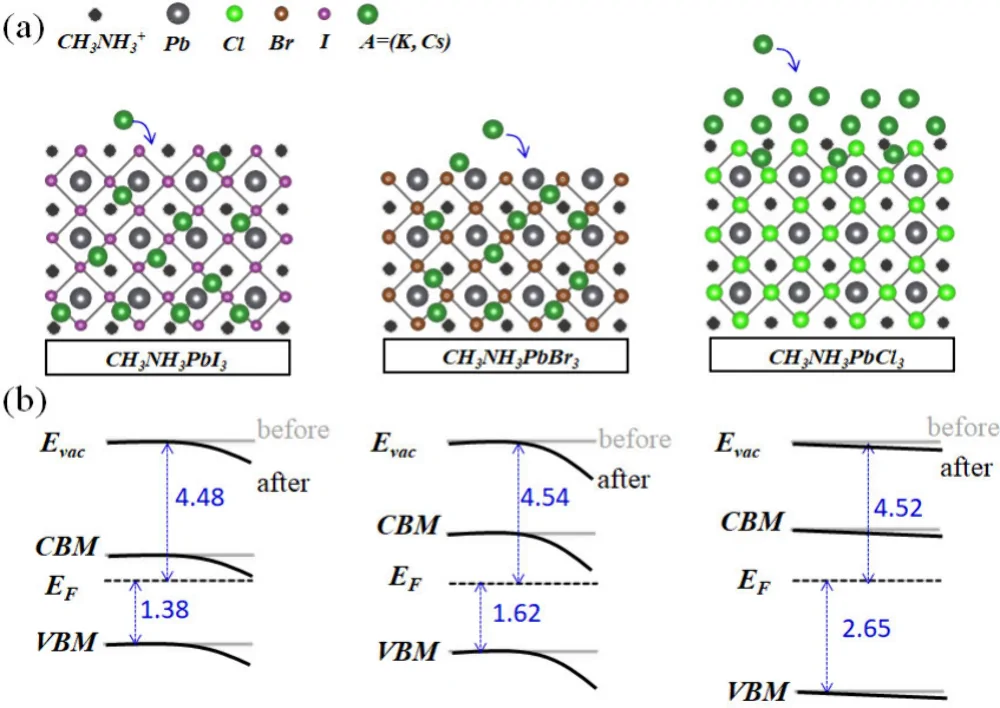
Schematic diagram of doping processes using alkali metals, giving the introduced alkali atoms diffusing into the bulk of perovskite single crystals and confining on the surface (a), leading to a varied behavior of the energy diagram change (b).

In summary, we systematically investigated the effects of alkali metal doping on CH_3_NH_3_PbX_3_ (X= I, Br, Cl) single crystals, focusing on interface energetics, temperature-dependent behavior, and band dispersions. Our results suggest that both K and Cs can readily diffuse into CH_3_NH_3_PbI_3_ and CH_3_NH_3_PbBr_3_ without disrupting crystalline structures. In contrast, despite their different ionic sizes, both alkali species remain strongly surface-enriched in CH_3_NH_3_PbCl_3_, consistent with kinetically hindered alkali migration in the chloride perovskite and, consequently, limited bulk doping. Temperature-dependent measurements further revealed different band-shift trends, highlighting that surface geometry and termination play a critical role in governing the evolution of the electronic structure in these perovskite single crystals. While this study is based on well-defined single crystals, the insights can extend to technologically important polycrystalline thin films and devices as doping directly tunes near-surface energetics, enabling improved energy-level alignment and lower resistance, more selective contacts. The observed diffusion and clustering behaviors provide a mechanistic basis for understanding alkali metal-induced band alignment and passivating defect states, reducing interfacial nonradiative recombination and associated voltage loss. Moreover, temperature-dependent behaviors may help explain performance variations and stability issues of perovskite material-based devices upon different environments. Therefore, doping is important not only for efficiency gains but also for improving the operational stability under realistic device conditions. Our findings will offer valuable guidance for tailoring growth processes, surface treatments, and interfacial engineering in device-relevant perovskite thin films.

## Methods

The CH_3_NH_3_PbX_3_ single crystals were prepared following methods proposed by Poglitsch and Weber.^42^ The crystal structure and bulk quality were initially characterized X-ray diffraction (XRD), with results summarized in Table S1. To obtain clean surfaces, larger single crystals were cleaved in an N_2_-filled glovebox, mounted on sample holders using conductive silver paste, and subsequently transferred into the ultrahigh-vacuum (UHV) apparatus for UPS measurements after overnight degassing under a substrate temperature of 350 K. The surface quality and cleanliness were verified prior to measurement using X-ray photoemission spectroscopy (XPS) (see Figure S9) and UPS. The results show no evidence of contamination or degradation and are in good agreement with previous studies,^19,40–45^ which report stable and no detectable contamination or degradation in polycrystalline perovskite thin films prepared by spin coating.^46–49^ UPS measurements were carried out using an ultralow-background, high-sensitivity hemispherical electron energy analyzer (MBS A-1) and monochromatic He I_α_ (*h*ν = 21.22 eV) radiation source.^50^ After the UPS measurements for confirming the surface cleanness on the perovskite single crystals, K and Cs metal atoms were deposited at 300 K from carefully outgassed dispensers (SAES Getters S.P.A.). During K and Cs deposition, the pressure in the preparation chamber remained below 1 × 10^–7^ Pa. The total thickness of K and Cs atoms are monitored by a quartz crystal microbalance. Using the angle-resolving mode of the analyzers, photoelectron-emission-angle (θ)-dependent ARPES measurements were performed in the respective acceptance angle ranges (36° for A-1) at the angular resolution of ∼0.34° for the A-1 system without changing the angular position of the analyzer against the surface normal. For the temperature-dependent UPS study, the perovskite crystal was slowly cooled from 300 to 100 K by using a combination of liquid helium and a resistive heater. The cooling rate was kept low at ∼0.42 K/min in order to minimize temperature-induced mechanical stress in the crystals.

## Supplementary Material



## Data Availability

Supporting data are available for this paper in Supporting Information. All other data supporting the plots within this paper and other findings of this study are available from the corresponding author upon reasonable request.

## Notes

CH_3_NH_3_PbX_3_ single crystals structure measured by using X-ray diffraction, XPS, UPS, MAES, and AFM results (PDF)
