# Supplementary material for: Evolution of the Electronic Structure of Hybrid Organic–Inorganic Halide Perovskite Single Crystals upon Alkali Metal Doping
Source: J Phys Chem Lett. 2026 Jul 29;17(32):9448–56. doi: 10.1021/acs.jpclett.6c01932 (PMC13488443; doi:10.1021/acs.jpclett.6c01932)
Supplement: Supplementary file 1 [file jz6c01932_si_001.pdf]

Supporting Information for

Evolution of Electronic Structure of Hybrid Organic–Inorganic Halide Perovskite Single Crystals upon Alkali Metal Doping

Jinpeng Yang[1], Tingyu Jiao[2], Wei Li[2], Bo Xu[3], Mats Fahlman[4], Satoshi Kera[5]* and Xianjie Liu[4]**

[1] College of Physical Science and Technology, Yangzhou University, Jiangsu, 225009, China

[2] National Key Laboratory for Metrology and Calibration Techniques, China Institute of Atomic Energy, Beijing 102413, China

[3] School of Science, China Pharmaceutical University, Nanjing, 211198, China

[4] Laboratory for Organic Electronics, Department of Science and Technology, Linköping University, Norrköping SE-60174, Sweden

[5] Institute for Molecular Science, Myodaiji, Okazaki 444-8585, Japan

Corresponding author:

E-mail: yangjp@yzu.edu.cn; kera@ims.ac.jp; xianjie.liu@liu.se

Table S1. Single crystal data for $CH_3NH_3PbX_3$ (X=I, Br, Cl)

Empirical Formula	CH6I3NPb (350K)	CH6Br3NPb (300K)	CH6Cl3NPb (300K)
**Formula Weight**	619.98	478.98	345.62
**Crystal Dimensions**	0.200 × 0.200 × 0.200 mm	0.200 × 0.200 × 0.200 mm	0.200 × 0.200 × 0.200 mm
**Crystal System**	cubic	cubic	cubic
**Lattice Type**	Primitive	Primitive	Primitive
**Lattice Parameters**	a = 6.29 Å	a = 5.90 Å	a= 5.67Å
	V = 248.86 Å^3	V = 206.24 Å^3	V = 187.72 Å^3
**Space Group**	P-43m (#215)	P-43m (#215)	P-43m (#215)
**Z value**	1	1	1

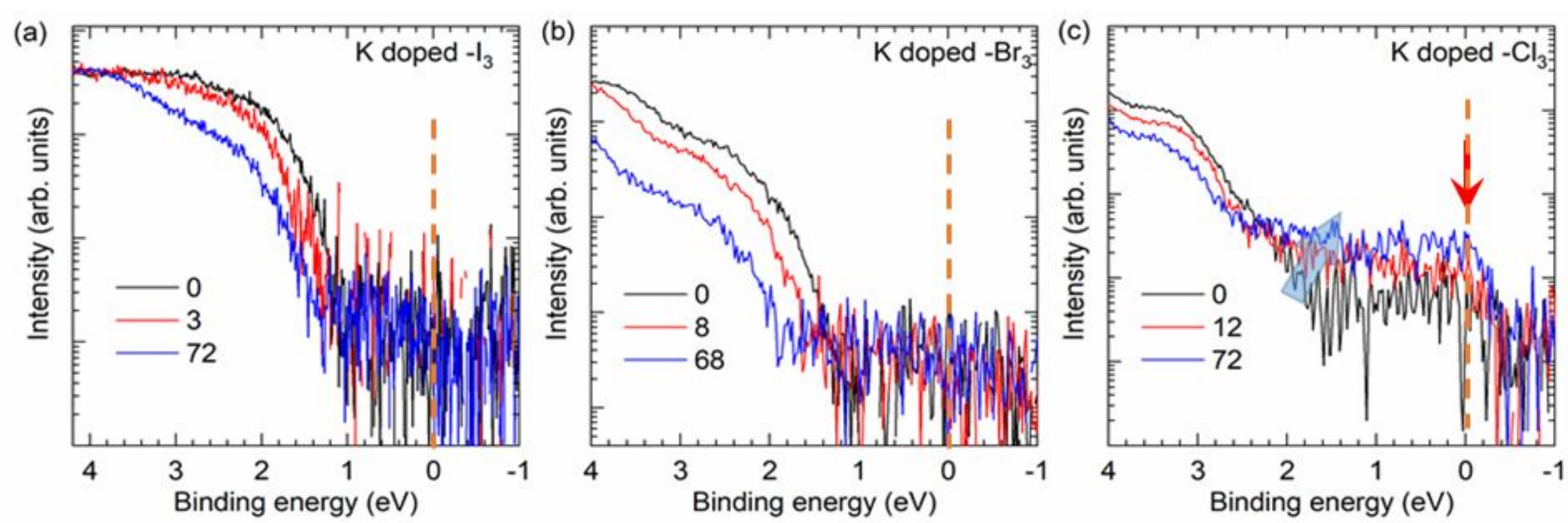

Figure S1. He I_α UPS spectra of (a)-(c) K-doped $CH_3NH_3PbX_3$ with X = I, Br, or Cl perovskite single crystal interfaces as a function of incremental deposited thickness with spectra plotted on a semi-logarithmic scale in the region near Fermi edge (vertical red dashed line) at the binding energy of 0 eV. The red arrow in (c) indicates the step-like feature at Fermi edge in K-deposited $CH_3NH_3PbCl_3$.

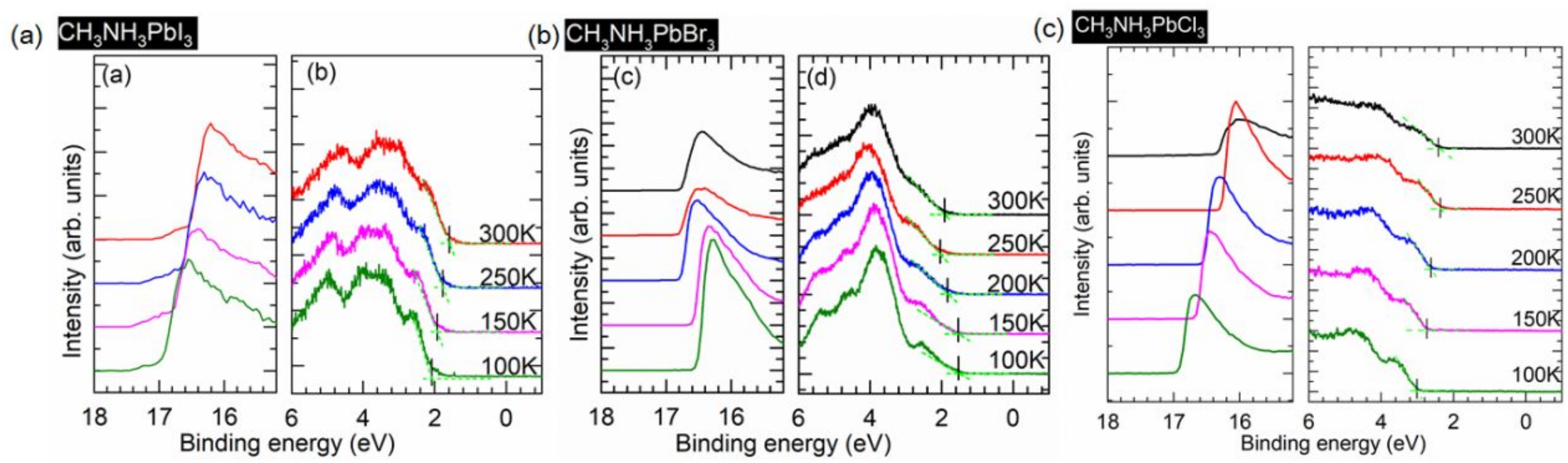

Figure S2. Temperature dependent UPS spectrum for pristine perovskite single crystals with (a) $CH_3NH_3PbI_3$, (b) $CH_3NH_3PbBr_3$, and (c) $CH_3NH_3PbCl_3$, respectively.

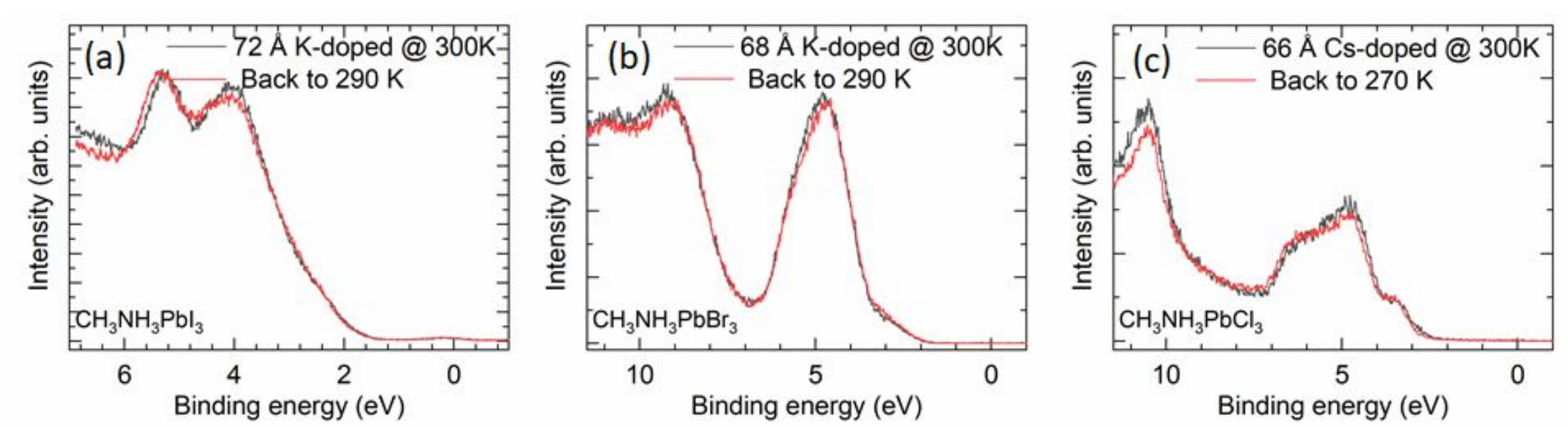

Figure S3. UPS spectra of alkali-metal surface-doped perovskite single crystals measured at 300 K, followed by low-temperature (100 K) measurements and subsequent warming back to near room temperature with (a) $CH_3NH_3PbI_3$, (b) $CH_3NH_3PbBr_3$, and (c) $CH_3NH_3PbCl_3$, respectively.

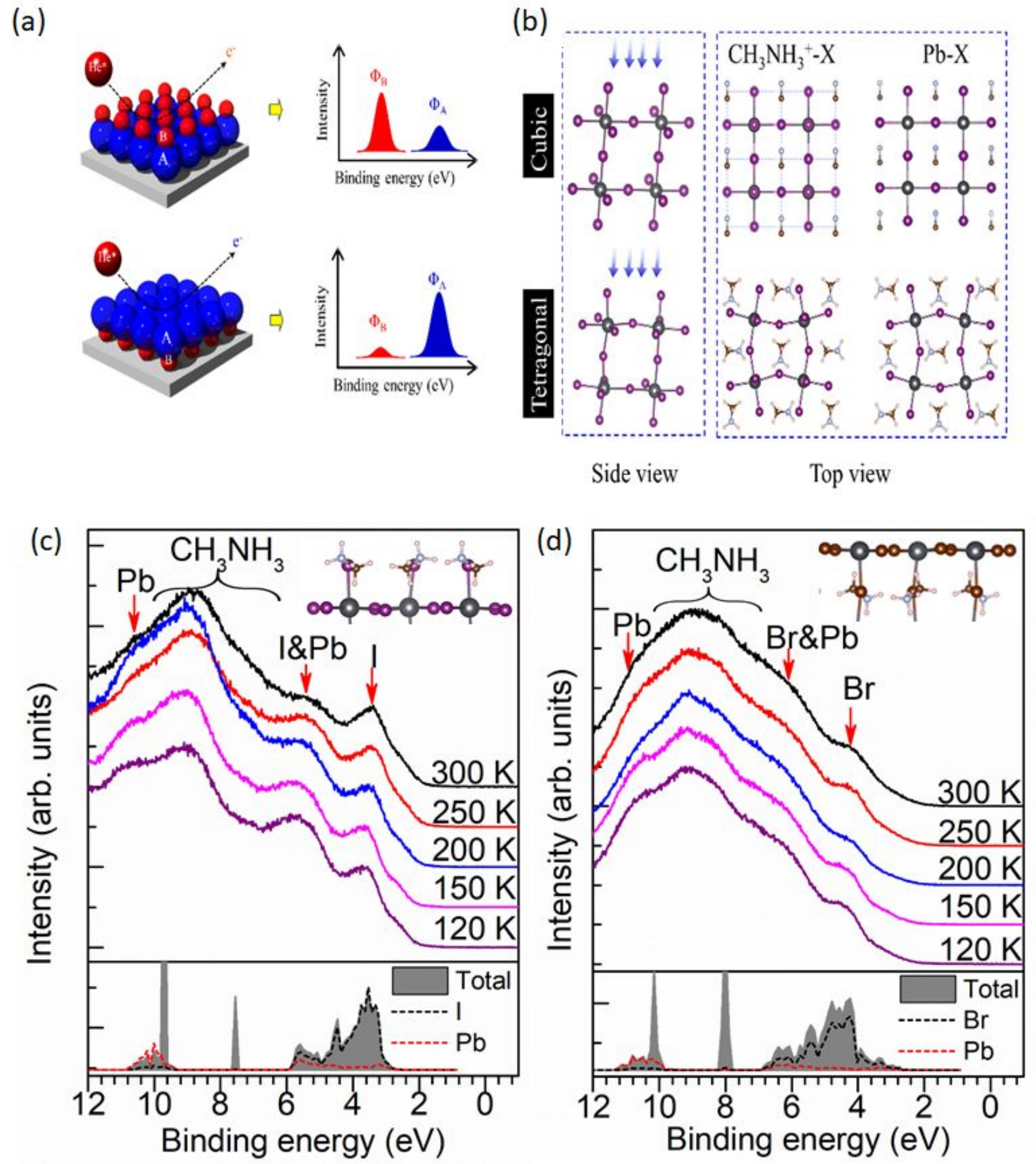

Figure S4. (a) The schematic diagram showing an ultrahigh surface sensitivity of MAES (metastable atom electron spectroscopy) through penning ionization to probe the topmost surfaces selectively, as large helium ion cannot penetrate beyond the surface. (b) Top and side views on $CH_3CH_3PbX_3$ perovskite single crystals with different topmost surfaces (CH_3CH_3-X or Pb-X) under cubic and tetragonal phases. (c)* MAES spectra of $CH_3NH_3PbI_3$ with temperature between 300 to 120 K where the dominant I 5p-related top feature is largely unchanged with cooling, while weaker Pb 6p-related features increase at lower temperature. It indicated surface termination is $CH_3NH_3^+$-I (A-X plane) compared with DFT-assisted valence-band assignments. (d)* MAES spectra of $CH_3NH_3PbBr_3$ between 300 to 120 K where the MAES line shape changes very little with temperature, indicating largely unchanged outermost-surface orbital distribution over this range with surface termination is Pb-Br (B-X plane) compared with DFT-assisted valence-band assignments.

* Yang, J.-P.; Meissner, M.; Yamaguchi, T.; Xi, B.; Takahashi, K.; Abdullah, S.; Liu, X.; Yoshida, H.; Fahlman, M.; Kera, S. Temperature-dependent band structure evolution determined by the organic cation dynamics in hybrid halide perovskites. Phys. Rev. B 2020, 102, 245101, DOI: 10.1103/PhysRevB.102.245101

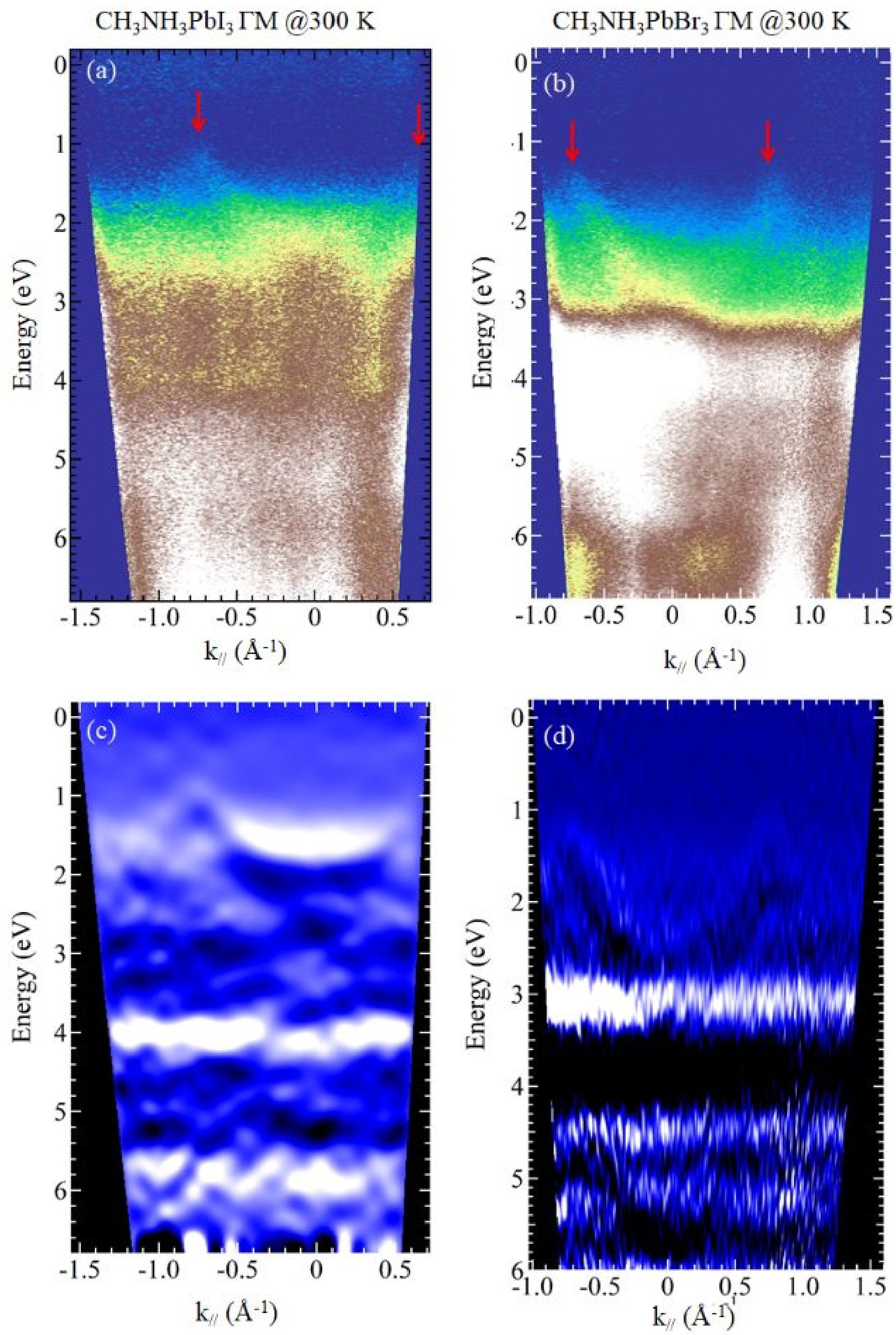

Figure S5. Mapping of E-$k_{//}$ dispersion along ΓM at 300K obtained from raw ARPES spectra in $CH_3NH_3PbI_3$ (a) and $CH_3NH_3PbBr_3$ crystals. (b) The corresponding second derivative of the measured ARPES intensity map ($-d^2I(E)/dE^2$) (c) $CH_3NH_3PbI_3$ and $CH_3NH_3PbBr_3$ (d). In all maps, note that there are reproducible band dispersions for the uppermost feature of the valence bands, which are simply easier to see in the processed data on the bottom, demonstrating the reliability of our $-d^2I(E)/dE^2$ maps.

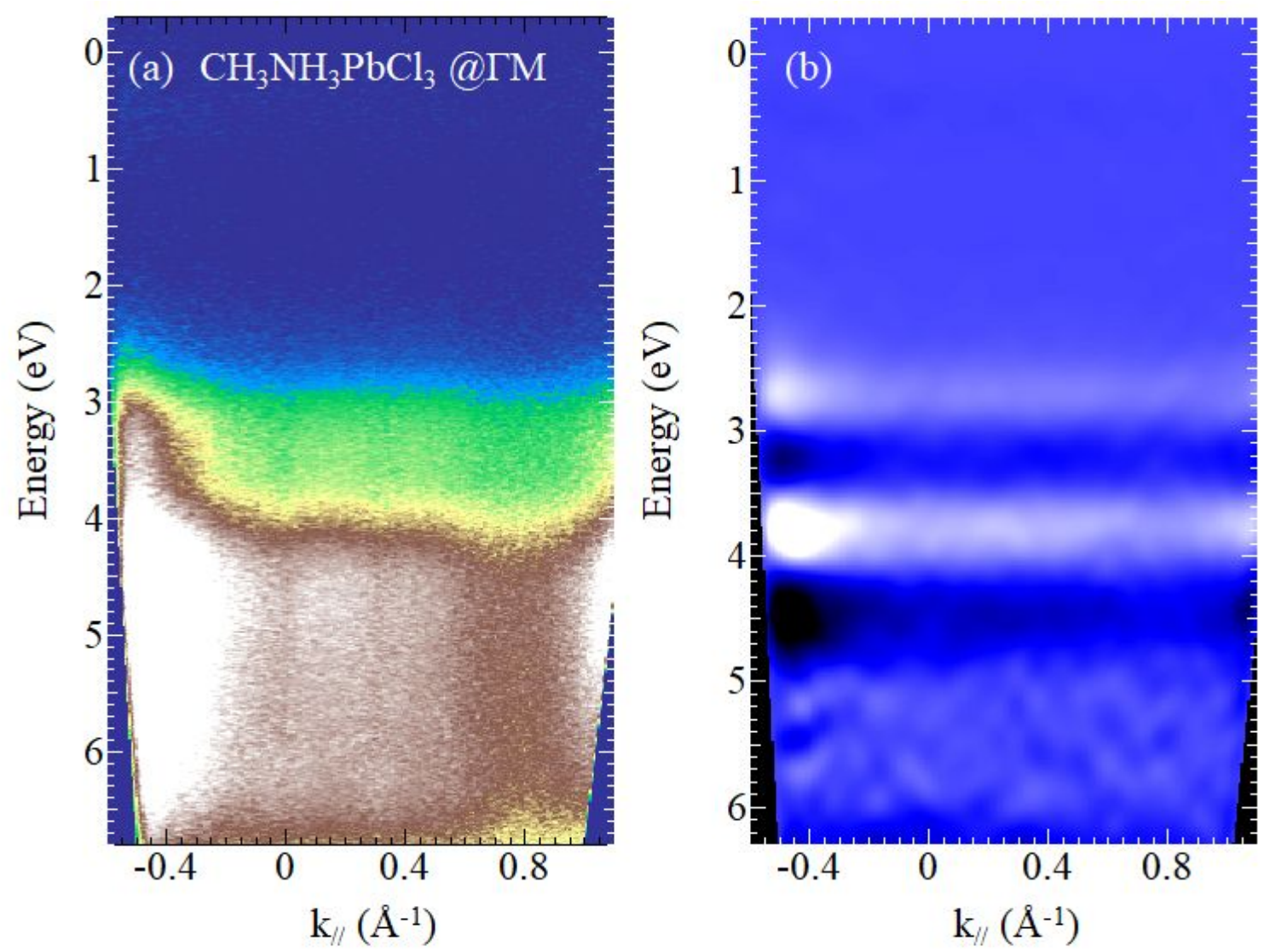

Figure S6. (a) E-$k_{//}$ dispersion maps of the $CH_3NH_3PbCl_3$ single crystal along the ΓM direction at 300 K, obtained from the raw ARPES spectrum. (b) the corresponding second derivative of the measured ARPES intensity map ($-d^2I(E)/dE^2$) map to enhance weak dispersive features. Notably, there is no clear band dispersion observed for the uppermost valence-band feature, which is attributed the small terraces surface of cleaved $CH_3NH_3PbCl_3$ single crystal surface.

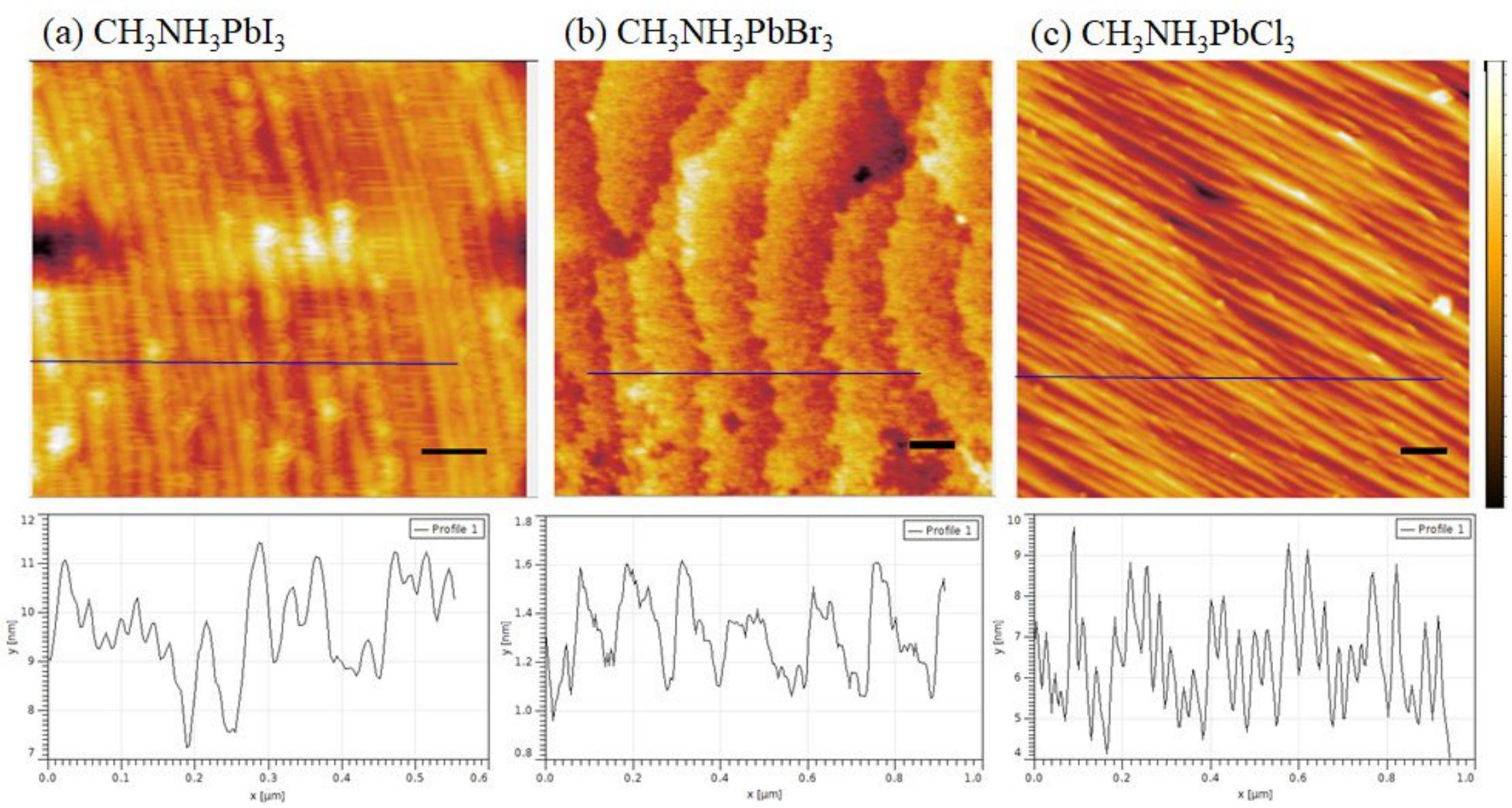

Figure S7. AFM images of the pristine perovskite single crystals $CH_3NH_3PbX_3$ after cleavage and line scan profiles. (a) $CH_3NH_3PbI_3$ and (b) $CH_3NH_3PbBr_3$ surfaces show sufficiently large, well-ordered terraces, but (c) $CH_3NH_3PbCl_3$ surface reveals only small flat regions with dense step bunching. The low parts are line scan profiles (blue line on each AFM image). The scale bar is 100 nm.

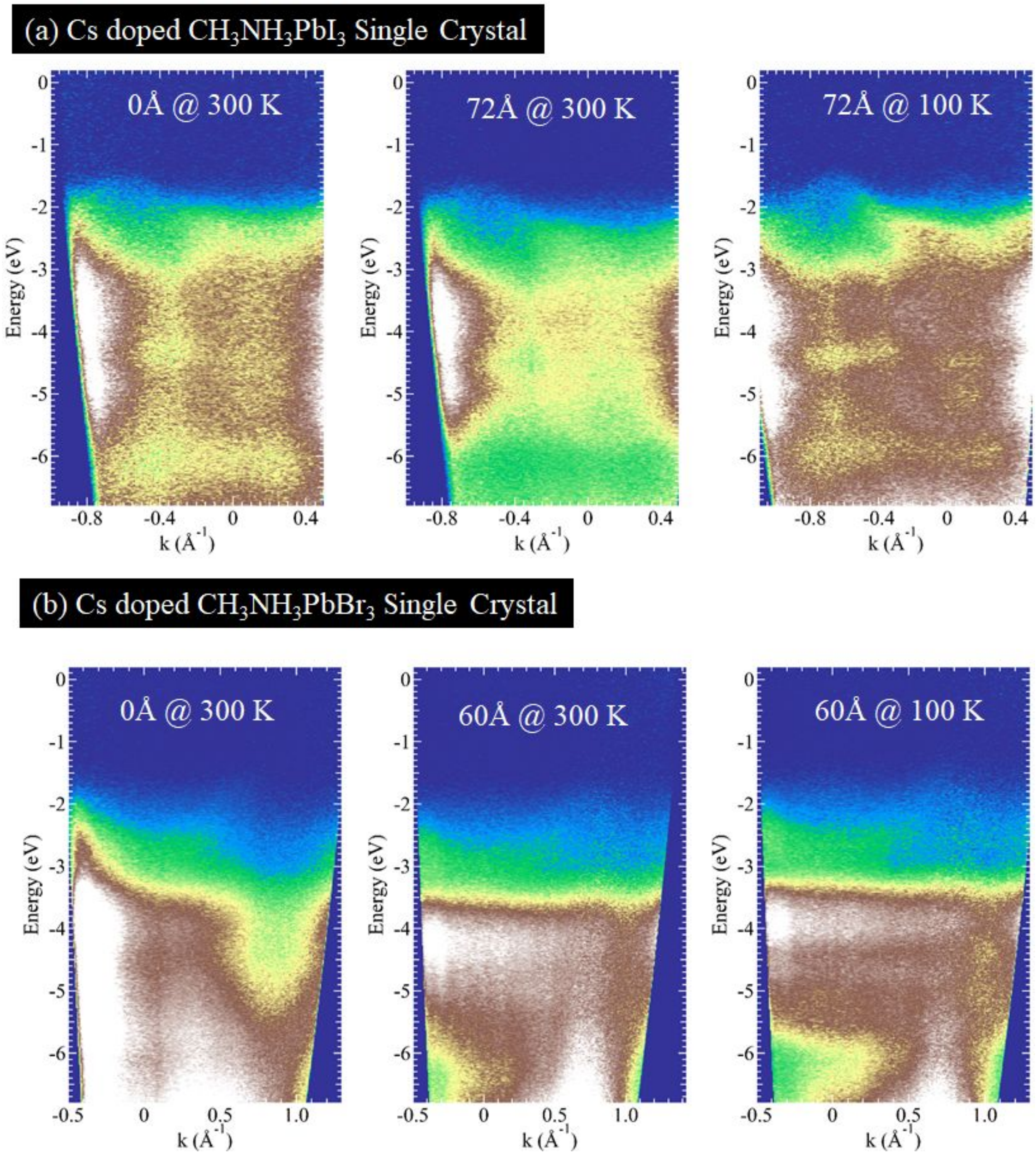

Figure S8. (a) Comparison of ARPES band mapping of $CH_3NH_3PbI_3$ along a cubic ΓM direction between pristine (0 Å) at 300 K, highest (72 Å) Cs doping at 300 K, and highest (72 Å) Cs doping at 100 K. (b) Comparison of ARPES band mapping of $CH_3NH_3PbBr_3$ along a cubic ΓM direction between pristine (0 Å) at 300 K, highest (60 Å) Cs doping at 300 K, and highest (60 Å) Cs doping at 100 K.

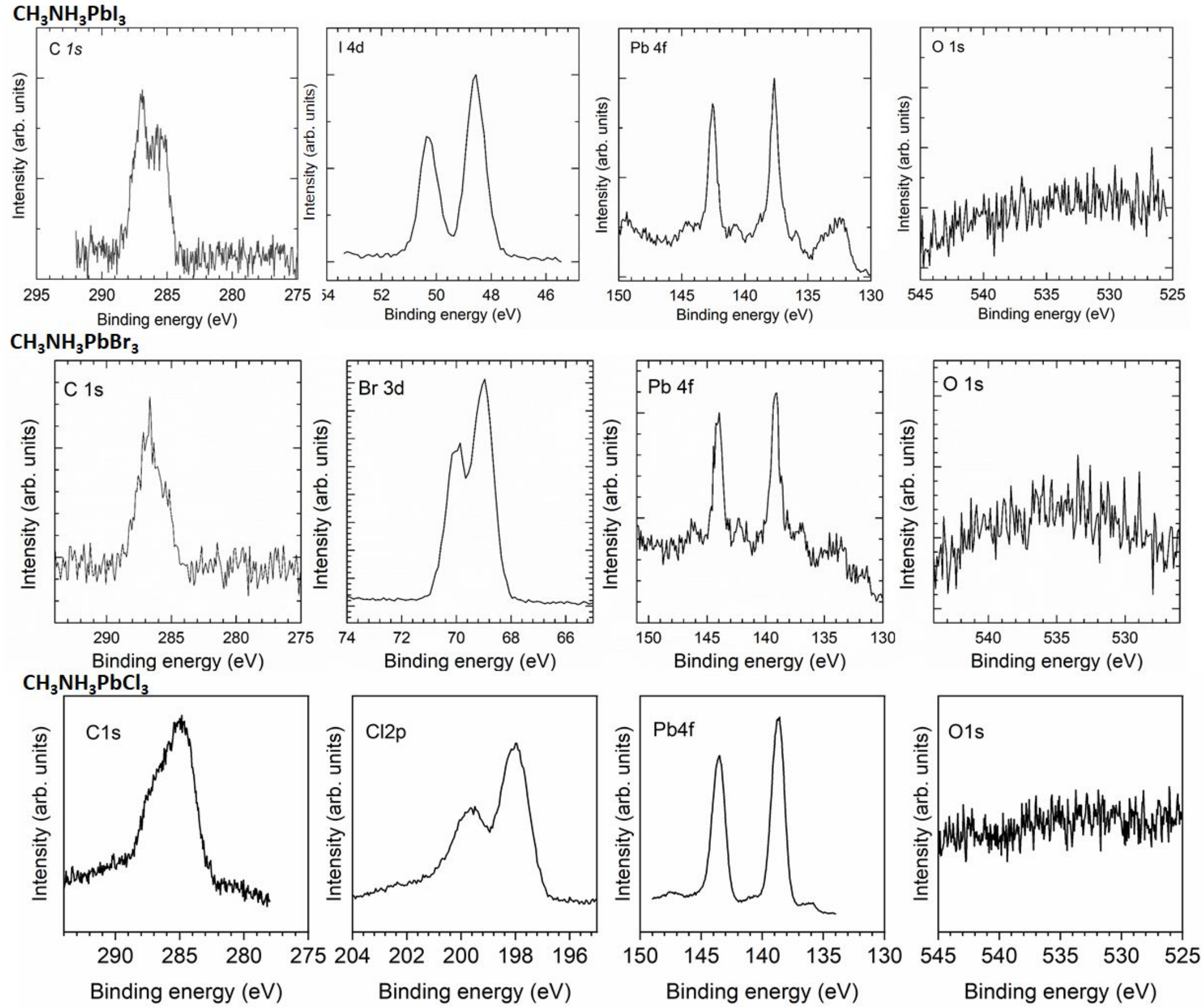

Figure S9. XPS spectra obtained from cleaved perovskite single crystals: $CH_3NH_3PbI_3$ (top), $CH_3NH_3PbBr_3$ (middle), $CH_3NH_3PbCl_3$ (bottom). In these XPS measurements, the samples were mounted without strict control of crystal orientation or surface flatness and were randomly attached onto silver paste with $CH_3NH_3PbI_3$ and $CH_3NH_3PbBr_3$ single crystals measured at FUNSOM, Suzhou, China, and the $CH_3NH_3PbCl_3$ single crystal checked at ITN, Norrköping, Sweden. It should be noted that no signal from oxygen can be detectable in each cleaved $CH_3NH_3PbX_3$ single crystal surface.
